# A dangerous food binge: a case report of hypokalemic periodic paralysis and review of current literature

**DOI:** 10.1186/s13052-022-01315-5

**Published:** 2022-07-15

**Authors:** Maria Carolina Colucci, Marica Fabiana Triolo, Simona Petrucci, Flaminia Pugnaloni, Massimiliano Corsino, Melania Evangelisti, Maria Cecilia D’Asdia, Giovanni Di Nardo, Matteo Garibaldi, Gianluca Terrin, Pasquale Parisi

**Affiliations:** 1 NESMOS department, Unit of Pediatrics, Sapienza University, c/o Sant Andrea University Hospital, Rome, Italy; 2 UOC Medical Genetics and Advanced Cell Diagnostics, Sant Andrea University Hospital, Rome, Italy; 3 Department of Clinical and Molecular Medicine, Sapienza University, Rome, Italy; 4 Division of Medical Genetics, IRCCS-Casa Sollievo della Sofferenza, San Giovanni Rotondo, Italy; 5 Department of Emergency Medicine, Sant Andrea Hospital University, Rome, Italy; 6 Department of Neuroscience, Mental Health and Sense Organs (NESMOS), Faculty of Medicine and Psychology, Sant Andrea Hospital University, Sapienza University, Rome, Italy; 7 Department of Neurology Mental Health and Sensory Organs (NESMOS), Unit of Neuromuscular Diseases, Neuromuscular Disease Centre, Faculty of Medicine and Psychology, Sant Andrea Hospital University, Sapienza University, Rome, Italy; 8 Department of Maternal and Child Health, Policlinico Umberto I, Sapienza University of Rome, Rome, Italy

**Keywords:** Periodic paralysis, Hypokalemia, Channelopathy, Adolescence

## Abstract

**Background:**

Hypokalemic periodic paralysis is a rare neuromuscular genetic disorder due to defect of ion channels and subsequent function impairment. It belongs to a periodic paralyses group including hyperkalemic periodic paralysis (HEKPP), hypokalemic periodic paralysis (HOKPP) and Andersen-Tawil syndrome (ATS). Clinical presentations are mostly characterized by episodes of flaccid generalized weakness with transient hypo- or hyperkalemia.

**Case presentation:**

A teenage boy presented to Emergency Department (ED) for acute weakness and no story of neurological disease, during the anamnestic interview he revealed that he had a carbohydrates-rich meal the previous evening. Through a focused diagnostic work-up the most frequent and dangerous causes of paralysis were excluded, but low serum potassium concentration and positive family history for periodic paralyses raised the diagnostic suspicion of HOKPP. After the acute management in ED, he was admitted to Pediatric Department where a potassium integration was started and the patient was counselled about avoiding daily life triggers. He was discharged in few days. Unfortunately, he presented again because of a new paralytic attack due to a sugar-rich food binge the previous evening. Again, he was admitted and treated by potassium integration. This time he was strongly made aware of the risks he may face in case of poor adherence to therapy or behavioral rules.

Currently, after 15 months, the boy is fine and no new flare-ups are reported.

**Conclusion:**

HOKPP is a rare disease but symptoms can have a remarkable impact on patients’ quality of life and can interfere with employment and educational opportunities. The treatment aims to minimize the paralysis attacks by restoring normal potassium level in order to reduce muscle excitability but it seems clear that a strong education of the patient about identification and avoidance triggering factors is essential to guarantee a benign clinical course. In our work we discuss the typical clinical presentation of these patients focusing on the key points of the diagnosis and on the challenges of therapeutic management especially in adolescence. A brief discussion of the most recent knowledge regarding this clinical condition follows.

## Main text

### Background

Periodic paralyses are rare inherited neuromuscular disorders included in the group of skeletal muscle channelopathies together with non-dystrophic myotonias. Channelopathies are due to mutations in genes encoding different ion channels with subsequent function impairment.

The periodic paralyses include hyperkalemic periodic paralysis (HEKPP, MIM#170500), HOKPP and Andersen-Tawil syndrome (ATS, MIM#170390) [1] .

Estimated prevalence of HOKPP is 1.12 per 100,000 live births [2]. Most cases of the HOKPP are hereditary or familial. They can be divided in HOKPP type 1 (HOKPP1, MIM#170400) caused by heterozygous mutations in the alpha 1S subunit of the skeletal muscle voltage-gated calcium channel gene CACNL1A3 (CACNA1S; MIM*114208), a 44 exons gene located on chromosome 1q32.1 encoding the L- type calcium channel (dihydropyridine receptor; DHPR), and the less common HOKPP type 2 (HOKPP2, MIM#613345) due to heterozygous mutation in the sodium channel gene SCN4A (MIM*603967), a 24 exons gene located on chromosome 17q23.3, encoding the voltage-gated sodium channel Nav1.4. To date, among all the pathogenic variants identified in these two genes, the most common voltage sensor mutations are the p.Arg528His, which was detected in our patient, and the p.Arg 672 His/Gly/Ser in CACNA1S, accounting about the 70–80% of mutated cases, and p.Arg669His and the p. Arg1539His in SCN4A, accounting a further 10%.

While sodium channels generate action potentials, DHPR is essential for excitation–contraction coupling. In both, channel mutations lead to the formation of an anomalous gating pore current that leads to unexcitability of sarcolemmal muscle, failure of muscle action potential generation, and subsequentially, flaccid paralysis attacks.

Periodic paralysis in HOKPP display a broad and heterogeneous clinical spectrum but are mostly characterized by episodes of flaccid generalized muscle weakness with decreased (seldom normal) deep tendon reflexes at physical examination and transient hypokalemia at blood test. Paralytic episodes are usually longer than in hyperkalemic periodic paralysis (several hours to days) and rarely respiratory and cardiac muscle impairment have been reported [3]. Weakness attacks usually occur in early teens during the night or in the early morning, being triggered by rest after strenuous exercise or carbohydrate rich meals consumptions. Also medications causing a dysregulation in serum potassium level may precipitate an attack. Patients are at increased risk for pre- or post-anesthetic paralysis and perioperative care including close control of plasma potassium concentration, avoidance of large glucose and salt loads, body temperature control, acid-base balance and careful use of neuromuscular blocking agents.

In patients with established family or personal history of HOKPP diagnosis is primarily clinical, otherwise the detection of a low serum potassium concentration during the attack supports diagnostic suspicion [4].

Other investigation, including thyroid function tests, potassium level dosage between the attacks, acid-base status, associated electrolyte disorders and electromyographic anomalies, allow to rule out causes of secondary periodic paralyses, such as thyrotoxicosis, renal tubular acidosis, Gitelman Syndrome or hyperaldosteronism, or other diseases that may mimic flaccid paralyses as Guillain-Barré syndrome, myasthenia gravis or spinal cord diseases [5].

Electrodiagnostic (EDX) studies are useful to rule out other mimicking neuromuscular conditions, while a supportive diagnostic test is represented by long-exercise test for muscle channelopathies among the EDX studies. During the paralytic attack, motor NCS can show a compound motor action potential (cMAP) reduction in about 70% of patients. Later a long exercise test can show different patterns of post-exercise reduction of amplitude of cMAP suggesting different types of periodic paralysis [6–8].

**Fig. 1:**
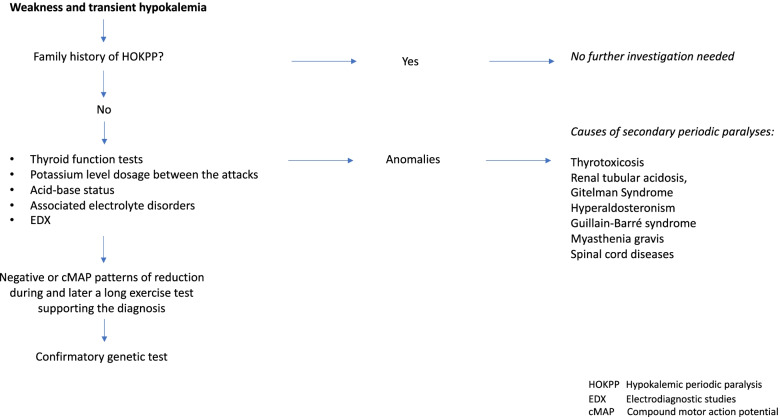
HOKPP Diagnostic Flowchart

Confirmatory genetic test is warranted, although in almost 20% of cases the genetic cause remains undefined. Provocative testing is potentially dangerous because it can precipitate life-threatening arrhythmias or hypoglycemia and therefore rarely practiced in pediatric settings (Fig. 1).

In patients with interictal muscle weakness muscle ultrasound and muscle MRI are reliable image techniques to reveal the possible fat replacement which can suggest a convergence of the phenotype towards a fixed myopathy [9].

Management of all these conditions aims to reduce the frequency of the paralysis attacks and long-term risk of muscular weakness. Treatment is mainly symptomatic and is based on restoring normal potassium level in order to reduce muscle excitability or modifying attack triggers [10]. Available pharmacological treatments are carbonic anhydrase inhibitors (CAI), that is acetazolamide and dichlorphenamide, historically employed in reducing weakness attacks frequency in both HOKPP and HEKPP. In order to get the best therapeutic strategy for each patient the first step is to identify triggering factors, especially dietary, and to strongly suggest lifestyle changes to minimize it.

In the light of the above, a multidisciplinary approach involving professional advice of a dietician may be useful. Symptoms can have a remarkable impact on patients’ quality of life and can interfere with employment and educational opportunities. It is crucial, therefore, to properly diagnose those disorders in order to establish a symptomatic treatment and impact on quality of life and morbidity.

There is poor scientific literature evaluating the effect of lifestyle changes on attacks and the aim of our contribution is to stimulate the scientific community to better investigate this aspect [11].

### Case presentation

A 14 years-old male presented at emergency department (ED) of Sant’Andrea Hospital in Rome in December 2020 for four limbs weakness acutely occurred in the night.

Upon arrival at ED the boy was alert and talkative, vital parameters were normal. On physical examination he showed generalized muscle weakness with diffusely reduced osteotendinous reflexes. No cranial nerves or autonomic dysfunction was detected.

**Fig. 2:**
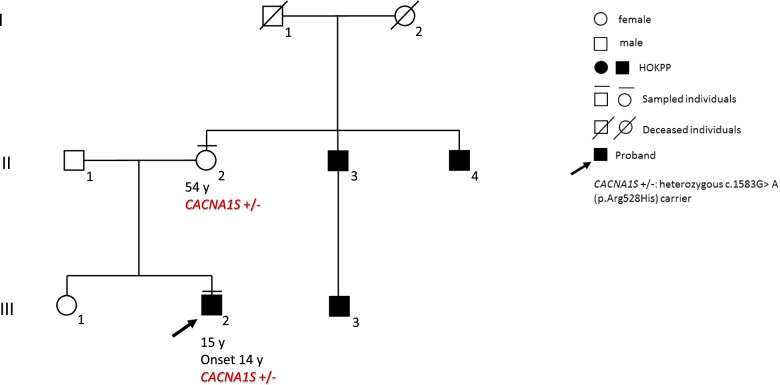
Patient family tree

Anamnestic interview revealed that he had a carbohydrates-rich meal the previous evening and that a similar episode occurred one week before with spontaneous resolution of symptoms within 2 hours. He was fully vaccinated and his personal medical history was unremarkable but he reported a familiarity for muscle weakness – never investigated – referred to two maternal uncles and one first cousin (Fig. 2).

Initial laboratory tests in ED showed mild hypokalemia (3 mmol/L; normal: 3.40–4.70 mmol/l) with normal other serum electrolytes, blood count, coagulation tests, renal function and liver function. Brain CT scan, cervicodorsal spine MRI and cerebrospinal fluid exam and colture were negatives.

Anamnestic history, clinical presentation and negative instrumental and laboratory test except of hypokalemia oriented us for a first episode of hypokalemic periodic paralysis (HOKPP, MIM # 170400). Therefore, we started on intravenous administration of potassium chloride, under electrocardiographic monitorization and we admitted him to the Pediatrics department. Symptoms progressively resolved and serum potassium value tested 12 hours later was normalized (4.40 mmol/L). Further investigation including CPK level, morning cortisol, ACTH, aldosterone, 24 hours urine collection, PTH, creatinine, thyroid function and abdomen ultrasound were performed in order to investigate the etiological nature of the disease. All these investigations were negative. EDX studies revealed normal sensory and motor nerve conduction studies (NCSs), needle electromyography (EMG) and short-exercise test for muscle channelopathies were normal. Conversely, the long-exercise test showed a progressive smaller cMAP up to about 40% of initial amplitude consistent with a pattern V of HOKPP. The diagnosis was genetically confirmed by the identification of the c.1583G > A (p.Arg528His) heterozygous variant in the CACNAS1 (RefSeq: NM_000069.3) gene by Next Generation Sequencing (NGS). The same variant was detected in the asymptomatic mother by Sanger sequencing. Unfortunately, segregation analysis could not be extended to the affected maternal relatives for poor compliance. The patient was discharged with oral potassium chloride supplementation and dietary and behavioral indication.

Unfortunately, one month later the boy came back to our attention because of a new episode of acute weakness. The evening before the onset of the symptoms he had a carbohydrate-rich meal and weakness occurred during physical activity. Physical examination was comparable to the previous access, he was unable to walk independently. Laboratory tests showed severe hypokalemia (1.60 mmol/L), without electrocardiographic anomalies. An intravenous potassium chloride infusion was started again and potassium values normalized after 30 hours (4.60 mmol/L) with consistent remission of the symptoms.

Dietary and behavioral rules were reiterated and the patient was discharged with an increased dosage of potassium chloride supplementation.

Currently, after 15 months, the boy is fine and no new flare-ups are reported.

## Discussion

Acute muscle weakness is a major neurological emergency in pediatrics and it is important to consider vital signs and respiratory symptoms, considering that some etiologies may be life-threatening and demand urgent care.

Neurological examination must address the distribution of weakness, impaired cranial nerves, and sensory and autonomic dysfunction.

Our diagnostic work-up was addressed by paralysis features (onset, localization, progression), associated symptoms (seizures, state of consciousness, fever) and by personal and family history of the patient. Considering differential diagnosis our patient underwent further investigations including neuroimaging tests and cerebrospinal fluid test in order to screen for other causes of paralysis but the personal and family history of our young patient, supported by the hypokalemia detected and by the absence of associated symptoms was strongly suggestive of a periodic paralysis.

Acute management of the patient involves immediate oral potassium supplementation or, secondarily, intravenous administration under close electrocardiographic monitoring because of the risk of life-threatening arrhythmias related to overcorrection of the serum potassium value. Pharmacological interventions consist of therapy to abort acute attacks and chronic preventive therapy to reduce attack frequency.

However, the real challenge in these patients is to prevent further paralyses and this can only be achieved by identifying and counselling the single patient on daily life triggers and making them aware of the risks they may face in case of poor adherence to therapy or behavioral rules.

This kind of intervention is made more difficult by the young age of our patients, especially in adolescence, so it is clear that the first reason for therapeutic failure is the lack of adherence.

## Conclusion

HOKPP is a rare disease but paralysis attacks can have a remarkable impact on patients’ quality of life and can interfere with employment and educational opportunities. The treatment aims to restore normal potassium level, reducing muscle excitability. It consists in oral potassium supplementation and a focused dietetic and lifestyle education. In our experience, patients should be managed by a multidisciplinary team, including a dietician and a psychologist and the single patient triggering factor should be identified making him aware of the risks he may face in case of poor compliance to therapy or behavioral rules. As it happens in others disease, adolescence is typically a phase of weak attendance to rules, and poor consciousness of health.

We therefore suggest that these patients deserve a stronger and more individualized work-up and we hope to stimulate scientific community to better investigate the mentioned aspects of this rare disease.

## Data Availability

Not applicable.

## Abbreviations

ATS: Andersen-Tawil Syndrome
CAI: carbonic anhydrase inhibitors
CMAP: compound muscle action
ED: emergency department
EMG: electromyography
FDA: Food and Drug Association
HEKPP: Hyperkalemic Periodic Paralysis
HOKPP: Hypokalemic Periodic Paralysis

## References

[1] Vivekanandam V, Munot P, Hanna MG, Matthews E (2020). Skeletal muscle Channelopathies. Neurol Clin.

[2] Fontaine B (2008). Periodic paralysis. Adv Genet.

[3] Kil TH, Kim JB (2010). Severe respiratory phenotype caused by a de novo Arg528Gly mutation in the CACNA1S gene in a patient with hypokalemic periodic paralysis. Eur J Paediatr Neurol.

[4] Phuyal P, Nagalli S (2021). Hypokalemic periodic paralysis.

[5] Statland JM, Fontaine B, Hanna MG (2018). Review of the diagnosis and treatment of periodic paralysis. Muscle Nerve.

[6] Fournier E, Arzel M, Sternberg D (2004). Electromyography guides toward subgroups of mutations in muscle channelopathies. Ann Neurol.

[7] Sharma CM, Nath K, Parekh J (2014). Reversible electrophysiological abnormalities in hypokalemic paralysis: case report of two cases. Ann Indian Acad Neurol.

[8] Zhang L, Niu J, Li Y, Guan Y, Cui L, Liu M (2020). Abduction range: a potential parameter for the long exercise test in hypokalemic periodic paralysis during inter-attack periods. Muscle Nerve.

[9] Weber MA, Jurkat-Rott K, Lerche H, Lehmann-Horn F (2019). Strength and muscle structure preserved during long-term therapy in a patient with hypokalemic periodic paralysis (Cav1.1-R1239G). J Neurol.

[10] Desaphy JF, Altamura C, Vicart S, Fontaine B Targeted Therapies for Skeletal Muscle Ion Channelopathies: Systematic Review and Steps Towards Precision Medicine *J Neuromuscul Dis* Published online December 7, 2020. doi:10.3233/JND-200582. 10.3233/JND-200582 PMC8203248 33325393

[11] Welland NL, Hæstad H, Fossmo HL, Giltvedt K, Ørstavik K, Nordstrøm M. The role of nutrition and physical activity as trigger factors of paralytic attacks in primary periodic paralysis. *JND* Published online February 24, 2021:1–12. doi:10.3233/JND-200604. 10.3233/JND-200604 PMC8385530 33646174

